# Administrative health data validity: Changes over 19 years

**DOI:** 10.23889/ijpds.v9i5.2634

**Published:** 2024-09-18

**Authors:** Jie Pan, Seungwon Lee, Cheligeer Cheligeer, Natalie Sapiro, Bing Li, Guosong Wu, Catherine Eastwood, Hude Quan, Yuan Xu

**Affiliations:** 1 Centre for Health Informatics, Cumming School of Medicine, University of Calgary; 2 Department of Community Health Sciences, Cumming School of Medicine, University of Calgary; 3 Lee Centre for Health Informatics, Cumming School of Medicine, University of Calgary; 4 Provincial Research Data Services, Alberta Health Services Libin Cardiovascular Institute, University of Calgary; 5 Libin Cardiovascular Institute, University of Calgary; 6 Department of Oncology, University of Calgary, Tom Baker Cancer Centre

## Objective

To evaluate validity of hospital-discharged abstract administrative health data (DAD) over years through chart reviews.

## Methods

We analyzed three chart review cohorts (4,008 patients in 2003, 3,045 in 2015, and 9,024 in 2022) in Calgary, Canada. Nurse reviewers determined the presence or absence of 17 clinical conditions employing similar protocols. The reviews were linked with DAD using a unique lifetime identifier, chart number, and admission date. We evaluated the validity of DAD, coded in ICD-10-Canada version, in recording conditions by comparing against chart reviews. The C-statistics was calculated in predicting in-hospital mortality.

## Results

The mean difference in prevalence between chart reviews and DAD for these 17 conditions was 2.1% in 2003, 7.6% in 2015, and 6.3% in 2022. However, some conditions were relatively stable, such as diabetes (1.9%, 2.1%, and 1.1%) and metastatic cancer (0.3%, 1.1%, and 0.4%). For 17 conditions, the sensitivity ranged 39.6-85.1% in 2003, 1.3-85.2% in 2015, and 3.0-89.7% in 2022. The C-statistics for in-hospital mortality based on DAD was 0.84 in 2003, 0.81 in 2015, and 0.78 in 2022.

## Conclusion

DAD increasingly under-coded conditions over 19 years. The validity of DAD decreased but remained relatively stable for certain conditions mandated for coding. The under-coding exerted minimal impact on in-hospital mortality prediction.

## Implication

The under-coding could be primarily due to the increase of hospital patient volumes and the limited time allocated to coders. Consequently, there is a need to develop artificial intelligence methods based on electronic medical records to support coding practices and improve coding quality.

